# Serum uric acid was an independent predictor of mortality in STsegment elevation myocardial infarction patients with Killip I other than Killip II-IV: Comment on "J Cardiovasc Thorac Res 2016;8:56-60"

**DOI:** 10.15171/jcvtr.2017.10

**Published:** 2017-03-06

**Authors:** Cheng-Wei Liu, Tzu-Chiao Lin

**Affiliations:** ^1^Division of Cardiology, Department of Internal Medicine, Tri-service General Hospital, Songshan Branch, National Defense Medical Center, Taipei, Taiwan; ^2^Graduate Institute of Clinical Medicine, National Taiwan University College of Medicine, Taipei, Taiwan; ^3^Division of Cardiology, Department of Internal Medicine, Tri-Service General Hospital, National Defense Medical Center, Taipei, Taiwan

Serum uric acid (SUA) was an independent predictor of mortality in ST-segment elevation myocardial infarction patients with Killip I other than Killip II-IV.

We read with much interest in Hajizadeh et al’s article that was recently published in the *Journal of Cardiovascular and Thoracic Research*.^1^ Hajizadeh et al enrolled acute STEMI patients undergoing thrombolytic therapies or primary percutaneous coronary interventions (PCIs); the higher SUA group was defined as a baseline value of SUA more than 8.0 mg/dL in male or 7.5 mg/dL in female, and Hajizadeh et al showed that a higher value of SUA was not associated with in-hospital and midterm mortality. However, several issues should be addressed. Firstly, the Hajizadeh et al’s study described too little information about their study population to clarify the association between SUA and mortality comprehensively; they did not report several vital data that interfered with outcome data in STEMI patients, including body mass index, smoking and infarct-related artery of baseline characteristics as well as door-to-balloon time, use of intra-aortic balloon pump and the successful rate of PCI procedures. Secondly, the rate of renal failure was significantly greater in the higher versus the lower SUA group. Both renal failure at baseline and contrast-induced acute kidney injury (in those patients undergoing primary PCI) were known to be associated with mortality. Thirdly, the severity of myocardial infarction, represented as an enzymatic myocardial infarct size, was significantly greater in the higher SUA group than in the lower SUA group. The higher SUA group of the Hajizadeh et al’s study had an almost doubled value of creatine phosphokinase (CPK) and a much greater value of CK-MB than in the lower SUA group. Given myocardial infarct size attributable to mortality, the rate of in-hospital death should be greater in the higher SUA group. Fourthly, outcomes were quite different in STEMI patients undergoing primary PCI from the patients undergoing thrombolytic therapies. Therefore, mixing these two types of therapeutic strategies of acute STEMI patients together without properly adjusting this critical confounder may result in an insignificant association among the SUA groups and mortality. Finally, a lower rate of in-hospital death in the higher SUA group that received fewer PCI procedures and had higher myocardial infarct size implied that higher SUA might have a protective effect against mortality. In a brief summary, Hajizadeh et al should re-evaluate the impact of aforementioned confounders on mortality and then the effect of SUA in the STEMI patients could be investigated fairly.

Hajizadeh et al showed the result that SUA was not associated with mortality consistent with other studies. In contrary, the abundant evidence suggested a significant association between higher SUA and mortality.^2-6^ The conflicting results could be explained by that heart failure severity as a complication of the STEMI patients, namely Killip’s classification, were as lower as approximately 20% in the Hajizadeh et al’s study than in other studies that mainly consisted of STEMI patients with Killip I undergoing primary PCI.^1-6^ In the PCI era, a higher value of SUA on admission was significantly associated with increased short- and long-term mortality in these studies.^5^ In our study, we could not either show a significant association between hyperuricemia and mortality in STEMI patients at Killip II-IV, but there was a significant association in the patients with Killip II-IV between hyperuricemia and 1-year mortality.^6^ Therefore, the distribution of Killip classification had a great impact on mortality when discussing an effect of SUA in STEMI patients. Actually, the interaction between hyperuricemia and Killip’s classification on mortality had been already pronounced in STEMI patients.^6^ We wondered that the result of Hajizadeh et al’s study would be changed if the authors methodologically correct selection bias and focus on the association between SUA and mortality in the Killip I STEMI patient who received primary PCI other than thrombolytic therapies. Conclusively, the relationship among SUA, Killip’s classification and mortality should be considered in STEMI patients, especially in the patients who underwent primary PCI.

## Competing interests

The authors have no conflict of interest.

## Ethical approval

Not applicable.
